# How Cold Shock Affects Ploidy Level and Early Ontogenetic Development of the Sterlet, *A. ruthenus* L.

**DOI:** 10.3390/ijms23010494

**Published:** 2022-01-01

**Authors:** Dorota Fopp-Bayat, Gyan Chandra, Anna Nitkiewicz

**Affiliations:** Department of Ichthyology and Aquaculture, Faculty of Animal Bioengineering, University of Warmia and Mazury in Olsztyn, 10-718 Olsztyn, Poland; gyanchandrakvk@gmail.com (G.C.); anna.nitkiewicz@uwm.edu.pl (A.N.)

**Keywords:** sturgeon, cold shock, polyploidization, parentage analysis, mosaic

## Abstract

The objective of the present research was to study the effect of cold shock (3 °C and 6 °C) on fertilized eggs of the sterlet, *Acipenser ruthenus* L. Cold shock was applied for various durations (30, 60 and 90 min) and the ploidy levels, survival, and genotypes of the treated embryos/larvae were recorded. Analysis of ploidy levels confirmed the presence of diploid, triploid, and mosaic (1n/2n, 2n/3n, and 1n/2n/3n) genotypes in experimental groups, while it was strictly diploid in control groups. Microsatellite genotyping confirmed both the incidence of polyspermy and retention of the 2nd polar body in experimental groups. However, patterns of inheritance in all diploid offspring in experimental and control groups revealed classical Mendelian disomic inheritance. Interestingly, the observed mosaic sterlets had normal morphology and were alive. However, some larvae had abnormal morphology which may be due to haploid syndrome. In all treatment groups (treatments: 3 °C–30 min; 3 °C–60 min; 3 °C–90 min; 6 °C–60 min), where the percentage of polyploid/mosaic larvae were high, the mortality was also high. Whereas, in the control groups (where there were only diploid (2n) larvae), the mortality was relatively low.

## 1. Introduction

Sturgeons are one of the most endangered groups of fish, widely exploited for expensive black caviar and tasty boneless meat [1,2,3]. Sterlet has a small body size and has one of the shortest reproductive cycles among sturgeons [4]. Sturgeons are classified into three ploidy groups: species with ~120 (*A. ruthenus*), ~240 (*A. baerii*), and ~360 (*A. brevirostrum*) chromosomes based on their DNA content [5]. Hybridization is common in sturgeon and is a severe threat to their endangered populations [6]. The natural populations of sturgeon are adversely affected by habitat destruction, blockage of spawning migration, pollution, and overexploitation [2,6]. The global aquaculture production of sturgeon biomass was about 102,327 tons, and caviar production was approximately 364 tons, in the year 2017 [7]. Therefore, studies on artificial breeding, embryonic developments, and factors affecting embryonic developments are essential for successful aquaculture and conservation of these valuable sturgeon resources.

The temperature of water bodies is the most important “key factor” influenced by various natural and anthropogenic factors, and it affects embryonic and larval developments, survival, and the recruitment process of sturgeon. Thus, it is described as the ‘abiotic master factor’ for fishes [8,9,10]. However, genetic characteristics, the developmental stage, and the thermal histories of species also decide the optimal temperature as well as the upper and lower lethal temperatures of a particular species, which vary between and among species [11,12]. At higher light intensity and temperature, the numbers of developmental abnormalities increase, while embryo viability decreases [13,14]. The early embryonic development phases of sturgeon are more sensitive to environmental factors than the subsequent phases [15]. Thus, information about the optimal temperature range for egg incubation is essential in successful fish larvae production, protection of fish species, and for the success of chromosome manipulation, which often requires a specific temperature regime [9,10,16,17]. Physical (pressure and temperature) or chemical shock at an early stage of embryonic development induce polyploidization, body deformities, disturbance in growth, and increased fish mortality [18,19]. The cold-shock treatment of fertilized eggs induces polyploidy (triploidy, tetraploidy, etc.) by the retention of the second meiotic polar body or by blocking the first mitotic division in the developing embryo [3,20]. In general, polyploid fish have a comparatively faster growth rate and larger body size as they convert food more efficiently and utilise more energy for somatic growth than reproduction, which is desirable in aquaculture and is in the interest of fish farmers [21,22,23]. In some cases, the eggs of polyploid loach (*Misgurnus aguillicaudatus*) are significantly larger than those of diploid individuals [24]. In salmonids, triploid fishes have a lower or similar growth rate in comparison to diploid counterparts [25,26,27].

In sturgeons and paddlefish, most researchers have applied the heat shock technique for triploidisation (retention of the second polar body) or diploidy restoration in meiotic gynogenesis [28,29,30,31,32,33,34,35,36,37,38], and very few researchers have applied cold shock techniques [39,40,41]. Heat shocks were applied for polyploidy induction in sturgeons, but the information about cold shock induction still needs to be expanded through more detailed studies. Reduced water temperature has sub-lethal and lethal effects on fish; it affects the fitness, physiology, and behaviour of fishes, and thus is considered a tool for fisheries science [42]. Furthermore, cold shocks are also observed under natural conditions due to rapid seasonal changes, precipitation, water movements, thermocline temperature variation, etc., but their consequences on fish populations are poorly understood [42]. Therefore, the main objective of the present research was to study the effects of cold shock (3 °C and 6 °C) on fertilized eggs for varying durations (30, 60, and 90 min) and observe the ploidy levels, survival, and genotypes of the treated embryos in the sterlet.

## 2. Results

**Figure 1:**
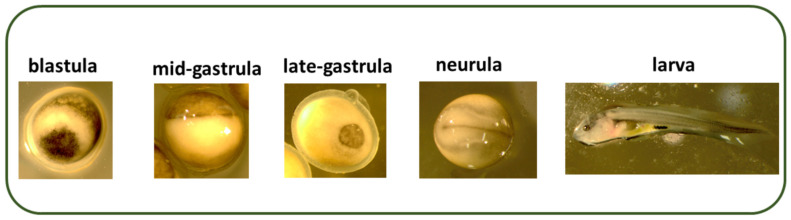
Different key embryonic stages (blastula, mid-gastrula, late-gastrula, neurula) and larva (with yolk sac) of sterlet, *Acipenser ruthenus*.

**Figure 2:**
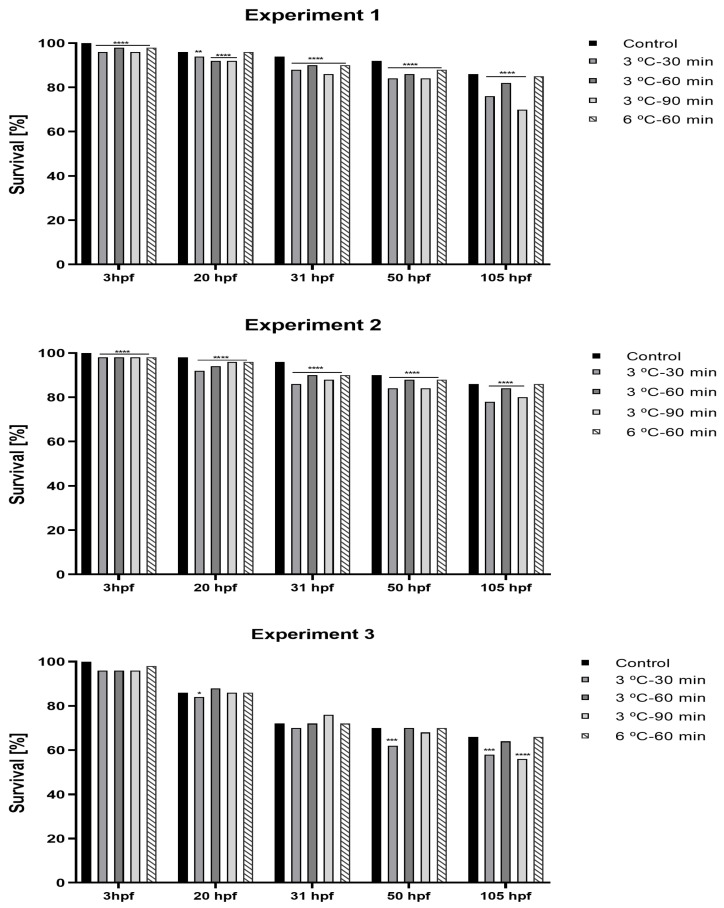
Percentage of survival of developing embryos (3 hpf, 20 hpf, 31 hpf, 50 hpf, 105 hpf; hpf—hours post fertilization) in experimental (E1, E2, E3) and control groups (C1, C2, C3) of sterlet, *Acipenser ruthenus* (asterisks denote the differences in the survival rate).

**Figure 3:**
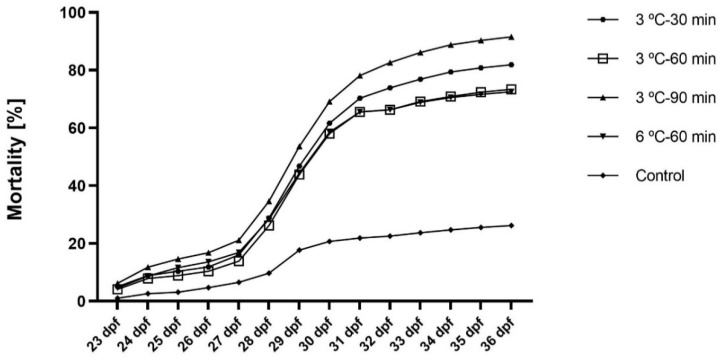
Survival of sterlet, *Acipenser ruthenus,* larvae during 36 days of experimental rearing.

**Figure 4:**
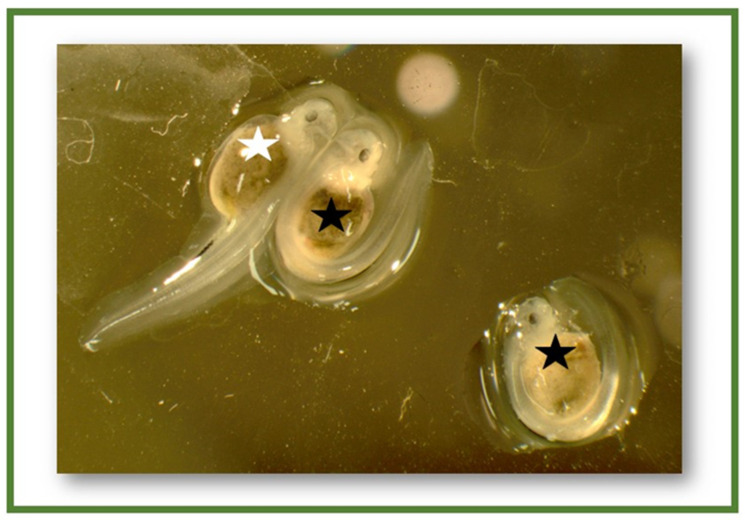
Normal and abnormal larvae of Sterlet, *Acipenser ruthenus* (white and black asterisks mark the normal and abnormal larvae, respectively).

The key embryonic stages (blastula, gastrula, and neurula) and larva (with yolk sac) are shown in Figure 1. Survival of embryos at fertilization, gastrulation, neurulation, and hatching stages (3–105 hpf) are presented in Figure 2. Hatching took place at 6 dpf, and high mortality occurred after yolk sac resorption (12 dpf) when larvae started exogenous feeding. The mortality of larvae during 23–36 dpf of the rearing period is shown in Figure 3. Larval mortality was higher in all treatments than in the control (Figure 3). Mortality was 26.2% in the control, whereas it was around 72.5% to 91.5% in treatment groups (Figure 3). In some larvae, the lethal effect was observed, which was similar to the haploid syndrome (Figure 4).

**Figure 5:**
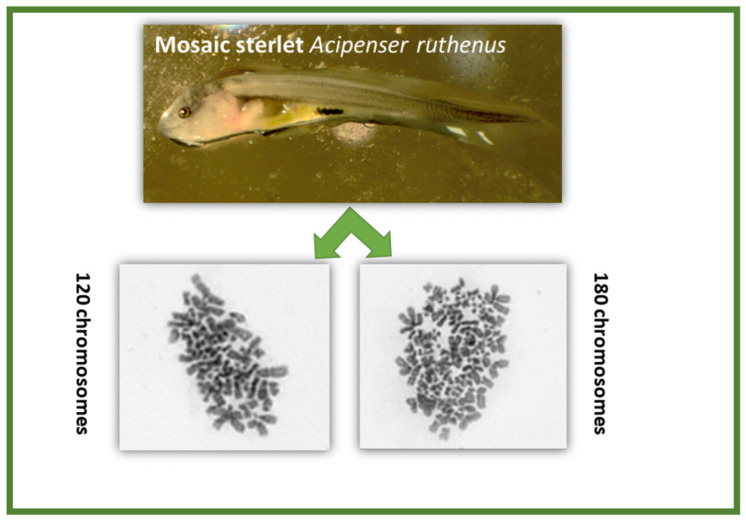
Mosaic (2n/3n) condition of larvae of sterlet, *Acipenser ruthenus*, shown by two different metaphase plates of a single individual.

Results of ploidy analysis revealed the presence of diploid, triploid, and mosaic genotypes (1n/2n, 2n/3n, and 1n/2n/3n) in experimental groups (E1, E2, and E3), while only diploid (~120 chromosomes) were observed in control groups (C1, C2, and C3) (Table 1). Two different metaphase plates observed in a single individual are presented in Figure 5, which indicates a 2n/3n mosaic condition. Parentage analyses were performed using a microsatellite marker, and observed genotypes are presented in Table 2. Microsatellite genotyping of both parental individuals and offspring revealed the incidence of polyspermy in one experimental group (E1) and retention of the second polar body in two experimental groups (E2 and E3), but not in control groups (C1, C2, and C3) (Table 2). Polyspermy is a condition where the genotype of triploid offspring has two alleles from the paternal origin and one from the maternal (Table 2; EI) and in retention of the 2nd polar body, the genotype of triploid offspring has two alleles from the maternal origin and one from the paternal (Table 2; EII, EII).

## 3. Discussion

The application of cold shock (3 °C and 6 °C) altered the ploidy levels, the pattern of inheritance, survival, and genotypes of the treated embryos in the sterlet. Our results also demonstrated that the mosaic genotypes of sterlets had normal morphology, and they were alive. In some haploid larvae, lethal effects were observed and characterized by abnormal morphology (haploid syndrome). The lethal effects may be because of an improper digestive tract, abnormality, or early asymmetry of cell division. Cold shock has been used for short-term anaesthesia [43], to change early sex ratios [44], and for induction of polyploidy (triploidy and tetraploidy) in fishes [45,46]. In the present study, these mosaic/polyploid sterlets could have been fertile or sterile. However, a conclusion on the reproductive capacity of mosaic/polyploid sterlets can be made only after the maturity of gonads. Diploid/triploid mosaic males of loach, *Misgurnus anguillicaudatus*, produced fertile diploid sperm, and females laid haploid, diploid, and triploid eggs simultaneously. Furthermore, triploid eggs were bigger [47,48].

### 3.1. Embryonic and Larval Developments, the Plasticity of Ploidy Level

In the present study, cold shock (3 ℃ and 6 ℃) induced three different kinds of mosaics (1n/2n, 2n/3n, and 1n/2n/3n) in the sterlet (Table 1). Similarly, cold-shock treatments on fertilized eggs resulted in the production of haploid, diploid, triploid, aneuploid, and mosaic (2n/3n and 1.3n/2.9n) individuals in loach [49]. Similar to the present study, other researchers also observed the presence of mosaic progenies during the triploidy induction [22,50,51]. Mosaic embryos can be identified at 2 to 4 cell stages of embryonic development. Mosaic embryos have a typical cleavage pattern and show a higher number of blastomeres at the 2 to 4 cell stages, with a characteristic mosaic haploid/diploid ploidy [52]. Larval mortality was low (26.2%) in the control group, but it was high (72.5% to 91.5%) in the treatment groups (Figure 3). The preponderance of mosaic/polyploid larvae in all treatment groups, as well as the preponderance of diploid larvae in the control groups, may be responsible for the high and low larval mortality, respectively (Table 1; Figure 3). Gheyas and Hussain [53] described a similar observation (survival) during triploidy induction in stinging catfish, *Heteropneustes fossilis*. In the present study, high mortality was observed after yolk sac resorption, which has been proven in many fishes [54,55]. However, higher mortality was observed in cold-shock-treated groups in comparison to the controls. Similarly, lower survival of triploid larvae was also observed by Chourrout et al. [56]. Triploidisation is the most common type of polyploidization that results in the formation of individuals with a triploid (3n) genome, and is characterized by the presence of an additional ½ genome—i.e., triploid sterlet have ~180 chromosomes [57]. Pressure shock techniques were more successful in retaining the second polar body in sturgeons [58,59]. Hydrostatic pressure shock is more effective than temperature shock in inducing triploidy, as in a closed hydrostatic pressure chamber, an equal amount of pressure is applied to all eggs, whereas all eggs do not receive an equal amount of shock in the temperature shock technique [60]. Recently, Flajshans et al. [59] applied a hydrostatic pressure shock (55 MPa) and heat shock (35 °C) to retain the second polar body (SPBR) for the production of triploid fish and restoration of diploidy during induced meiotic gynogenesis (MeiG) in sterlet. During the experiment, hydrostatic pressure shock provided the highest fertilization rate (62.14 ± 8.90%) and hatching rate (24.59 ± 8.35%).

Alcantar-Vazquez applied cold shock in spotted sand bass (*Paralabrax maculatofasciatus*) to induce triploidy, and found a significantly higher number of abnormal cell cleavages during embryogenesis, while the hatching rate, survival, and the proportion of live yolk-sac larvae were significantly lower, which correlated with abnormal cell cleavage [61]. However, in Atlantic cod (*Gadus morhua*), which inhabit cold waters, cold shock did not cause abnormal cell cleavage during embryonic development [62]. The temperature difference between the maintenance temperature and the cold-shock temperature is pivotal as it determines the effects of shock in developing embryos and larvae, including abnormal cleavage and abnormalities [63]. For instance, the effect of cold shock in terms of abnormal cleavage was significant in spotted sand bass, where the difference was 14 °C. In contrast, it was not significant in Atlantic cod, where the difference was only 5 °C [61]. However, in the present study, the temperature differences were 13 °C and 10 °C and thus could cause abnormal cleavage. In addition, there is a possibility that the mosaicisms were observed because of asymmetrical cell divisions. Iegorova et al. also observed atypical division (AD) in embryos of sturgeons, and found that all AD embryos were mosaic with normal morphology; survival of AD embryos ranged from 28.6% to 100% at the 7 DPF stage (hatching), while it was 44.3–86.5% in control groups [52].

### 3.2. Segregation of Alleles and Parentage Analysis

The offspring from experiments (E1) had alleles (234 and 270 bp) that were inherited paternally at locus *Spl-106* and supported the phenomenon of polyspermy (Table 2). Polyspermy is a condition where an egg is fertilized by more than one sperm. It has been described in sturgeon [52], as the oocytes of sturgeons have multiple micropyles [64]. On the contrary, offspring from experiments (E2 and E3) had alleles (313 and 243 bp; 286 and 278 bp; 204 and 212 bp) that inherited maternally at loci *Spl-106* and *Spl-163*; this indicated the retention of the second polar body which may be induced by cold shock (Table 2). However, patterns of inheritance in all diploid offspring in experimental and control groups demonstrated classical Mendelian disomic inheritance (Table 2). The present findings also suggest that cold shock promotes the retention of the second polar body as well as polyspermy in sterlet. The anomalies in ploidy levels of the offspring in the experimental groups indicate the extraordinary reproductive and developmental plasticity of sturgeons that inherit a double maternal or paternal genome in polyploid descendants. The findings of the present study reveal the impact of cold shock on the genome of sturgeons, which is prone to polyploidization events. Null alleles were evident in both parents that appeared to be homozygous at particular microsatellite loci (*Spl-106*) and caused an interpreting error in parentage analysis [65] (Table 2). Further research is essential to find the robust molecular markers that can identify the maternal and paternal inheritance in the duplicated genome, particularly in sturgeon where null alleles are more common.

This study successfully demonstrated that cold shock could alter the ploidy levels (triploid), the pattern of inheritance (polyspermy and retention of second polar body), survival, and genotypes (mosaicism) in sterlet. The ploidy level and genetic makeup of stocking materials must be considered since it may cause much more serious problems in the aquaculture and ranching programme of sturgeon. The viability of mosaic sterlets is very interesting not only for scientists but also for hatchery managers—especially where there is a possibility of the presence of such mosaic specimens in the spawning stocks. Will these mosaic/triploid offspring be fertile or sterile? That is an important question whose answer will be very helpful in understanding the reproductive biology and physiology of valuable and troubled sturgeon. It will be the next phase of the investigation.

## 4. Materials and Methods

### 4.1. Controlled Reproduction with Cold Shock

**Figure 6:**
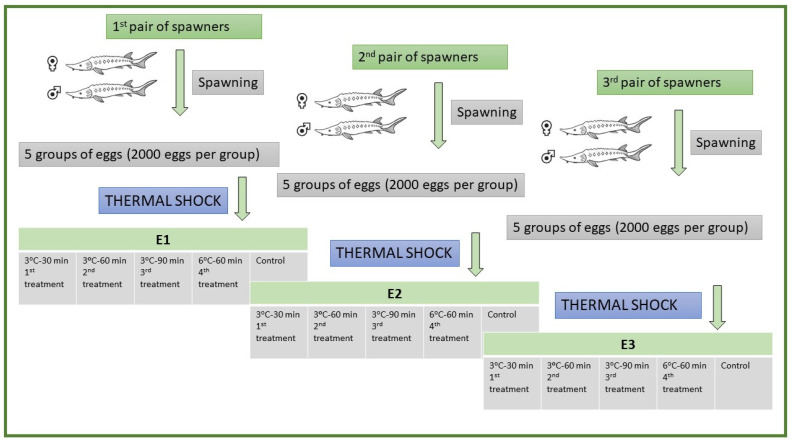
Flow diagram of the experiment with the three different pairs of sterlets used in the study.

We conducted experimental reproduction on three females and three males of sterlet (Figure 6). Females were injected with pituitary extract of *Cyprinus carpio* at the rate of 5 mg kg^−1^ body weight, whereas males were injected with a single dose of Ovopel (mammalian GnRH analogue + metoclopramide) at the rate of 1 g kg^−1^ bodyweight for spawning and spermiation, respectively [66]. Then, injected males and females were transferred separately into a recirculating water system at a temperature of 15 °C. Stripping was done for the collection of eggs and milt was collected using a syringe. We divided the eggs of each female into five portions (four treatments: 3 °C–30 min; 3 °C–60 min; 3 °C–90 min; 6 °C–60 min; and one control) in such a way that each portion had 2000 eggs (Figure 6). We diluted milt in the water (1:50 ratio) and fertilized the eggs; activated eggs were either cold-shocked (treatments) or not (control). After 5 min post eggs’ activation, activated eggs were subjected to cold shock of 3 °C for the duration of 30, 60, or 90 min. We also applied a cold shock of 6 °C at 5 min post eggs’ activation for 60 min. We calculated the percentage of fertilization at 3 h post fertilization (hpf) and recorded the survival of embryos at the middle of gastrulation, the end of gastrulation, neurulation, and hatching in each treatment (3 °C–30 min; 3 °C–60 min; 3 °C–90 min; and 6 °C–60 min) and control. We repeated this experiment three times and named them E1, E2, and E3, respectively, and used three pairs of brooders for three experiments (E1, E2, and E3)—i.e., one pair for one experiment (Figure 6).

### 4.2. Embryo Incubation and Experimental Larval Rearing

We transferred both cold-shock-treated (3 °C and 6 °C) and untreated eggs into the experimental cage incubators and maintained the temperature of the thermoregulated incubation system at 15 °C. The quality of ova was checked in both the treatment and control groups. After hatching, we transferred larvae into a special system of aquaria and maintained the temperature at 16 °C. We observed the larval development and survival during the rearing period (until 36 days post-fertilization (dpf)). In the beginning, we reared larvae in 5 experimental aquaria in a RAS system (16 °C) and maintained the photoperiod at 12 h:12 h; we initiated larval feeding at 6 days post-hatch (dph) after resorption of the yolk sac, and larval mortality was recorded every day in the morning. First, we used live nauplii of *Artemia* sp. (GSL origin, INVE Aquaculture, Belgium) for feeding (from 13 dpf) and then formulated starters (Perla Larvae Proactive, Skretting, Norway) were offered three times a day. During the incubation period of 24 hpf to 120 hpf, we sampled embryos from each treatment (3 °C–30 min; 3 °C–60 min; 3 °C–90 min; and 6 °C–60 min) and the control. Sampled embryos were observed for embryonic development at the main development stage (fertilization, mid-gastrula, late-gastrula, neurula, and larvae). We observed the embryonic and larval developments in a Olympus KL2500 LCD stereoscope microscope (Tokyo, Japan); we identified the embryonic stages in experimental and control groups as described by Dettlaff et al. [4] in Russian sturgeon, *Acipenser gueldenstaedtii*. Photographs were taken and compared with similar embryonic stages of development as described in Russian sturgeon [4]. Furthermore, all analyzed embryonic stages were also compared with the same development stages of the control groups.

### 4.3. Ploidy Analysis of Experimental Larvae

We randomly sampled ten larvae from each treatment and control group and placed them in a 0.025% solution of colchicine for 4 h at room temperature (16 °C). Larvae were alive in the colchicine solution. The heads of larvae were dissected and hypotonized in 0.025% KCl for 45 min (in the refrigerator). Then, they were fixed in methanol/acetic acid 3:1 solution three times (in the refrigerator): the first time for 30 min and then two times for 15 min. The fixed heads were homogenized in Eppendorf tubes and the cell suspensions were placed in a microscope slide. Dried chromosome preparations were stained with 5% Giemsa solution for 20 min. We analysed at least ten good-quality metaphase spreads from each cytogenetically studied larva under a Zeiss Axio Imager A1 microscope equipped with a fluorescent lamp and a digital camera; captured images were processed electronically using Band View/FISH View software (Applied Spectral Imaging, Carlsbad, CA, USA). The number of chromosomes was counted in all treatment and control groups for ploidy estimation.

### 4.4. Survival of Embryos and Larvae in Experimental Rearing

We removed dead embryos systematically and calculated the percentage of fertilization at 3–4 hpf during first or second cell division. We recorded the survival of developing embryos and larvae at different developmental stages, that is, fertilization (3 hpf), neurulation (31–50 hpf), and hatching (6 hpf) (Figure 2). After hatching, abnormal larvae and unhatched eggs were counted and carefully removed from the tank. At 6 dpf, free-swimming larvae were counted, and the hatching rate of larvae (%) was calculated in each treatment and control group. At 12 dpf, high larval mortality was observed when larvae started exogenous feeding, and we decided not to include this part of rearing as it is very common in fish development. At 15–16 dpf, we observed a decreasing trend in mortality and hence, mortality of larvae from 23 dpf to 36 dpf was recorded (Figure 3).

### 4.5. Molecular Analysis

Fin clips were collected from each parental individual (3 females and 3 males) and stored in 96% ethanol for genetic analysis. We randomly selected 30 larvae from each treatment and control group on 6–10 dpf, and stored them in 96% ethanol. We extracted genomic DNA using the Sherlock AX kit (A&A Biotechnology, Poland, Gdansk) and amplified two microsatellite loci: *Spl-106* and *Spl-163* [67] following Fopp-Bayat [68] and Fopp-Bayat and Ocalewicz [69]. We analyzed the amplified DNA fragments using an Applied Biosystems 3130 Genetic Analyzer sequencer against GeneScan 500 [LIZ] size standard. Individual microsatellite loci were amplified using primers with different attached fluorescent dyes (FAM, NED, Life Technologies, California, USA), arranged into sets, and analyzed in multiplex mode. Analyses of microsatellite DNA fragments were performed using GeneMapper software and Genetic Analyzer software, following the manufacturers’ instructions.

## Figures and Tables

**Table 1:** Observed plasticity of ploidy level in cold-shock-treated offspring of sterlet, *Acipenser ruthenus*.

Experiment	Experimental Groups	Ploidy Variants (%)				
		1n/2n	2n/3n	2n	3n	1n/2n/3n
EI	3 °C–30 min	20	-	-	-	80
	3 °C–60 min	-	70	5	25	-
	3 °C–90 min	-	80	9	11	-
	6 °C–60 min	-	8	92	-	-
	Control	-	-	100	-	-
EII	3 °C–30 min	30	-	-	-	70
	3 °C–60 min	-	70	10	20	-
	3 °C–90 min	-	80	10	10	-
	6 °C–60 min	-	7	93	-	-
	Control	-	-	100	-	-
EIII	3 °C–30 min	-	-	-	-	100
	3 °C–60 min	-	100	-	-	-
	3 °C–90 min	-	100	-	-	-
	6 °C–60 min	-	8	92	-	-
	Control	-	-	100	-	-

**Table 2:** Segregation of alleles (in base pairs) at two microsatellite loci in cold-shock-treated offspring (diploid, triploid, mosaic) of sterlet (*Acipenser ruthenus*) polyspermy is indicated by the dotted line (-------) and retention of second polar body is indicated by a straight line (—); the numbers of observed genotypes are mentioned in parenthesis.

Experimental Groups	Locus	Observed Genotype								
		Males—Spermatozoa Donors			Females—Oocyte Donors			Offspring from Experimental Groups		
		EI	EII	EIII	EI	EII	EIII	EI	EII	EIII
3 °C–30 min	*Spl-106*	234/270	234/270	260	313	313/243	286/278	234/313 (12)	234/313 (7)	286/278/260 (30)
								270/313 (16)	234/243 (4)	
								234/270/313 (2)	270/313 (11)	
									270/243 (2)	
									234/313/243 (2)	
									270/313/243 (4)	
	*Spl-163*	188/204	188/204	188/196	188/204	188/204	204/212	188/204 (30)	188/204 (30)	212/204/196 (16)
										212/204/188 (14)
3 °C–60 min	*Spl-106*	234/270	234/270	260	313	313/243	286/278	234/313 (13)	234/313 (5)	286/278/260 (30)
								270/313 (15)	234/243 (3)	
								234/270/313 (2)	270/313 (8)	
									270/243 (1)	
									234/313/243 (6)	
									270/313/243 (7)	
	*Spl-163*	188/204	188/204	188/196	188/204	188/204	204/212	188/204 (30)	188/204 (30)	212/204/196 (15)
										212/204/188 (15)
3 °C–90 min	*Spl-106*	234/270	234/270	260	313	313/243	286/278	234/313 (13)	234/313 (6)	286/278/260 (30)
								270/313 (16)	234/243 (2)	
								234/270/313 (1)	270/313 (8)	
									270/243 (1)	
									234/313/243 (7)	
									270/313/243 (6)	
	*Spl-163*	188/204	188/204	188/196	188/204	188/204	204/212	188/204 (11)	188/204 (10)	212/204/196 (16)
								188/188 (9)	188/188 (9)	212/204/188 (14)
								204/204 (10)	204/204 (11)	
6 °C–60 min	*Spl-106*	234/270	234/270	260	313	313/243	286/278	234/313 (13)	234/313 (8)	286/260 (15)
								270/313 (16)	270/313 (12)	278/260 (13)
								234/270/313 (1)	234/243 (4)	286/278/260 (2)
									270/243 (3)	
									234/313/243 (1)	
									270/313/243 (2)	
	*Spl-163*	188/204	188/204	188/196	188/204	188/204	204/212	188/204 (9)	188/204 (9)	188/204 (7)
								188/188 (10)	188/188 (9)	188/212 (6)
								204/204 (11)	204/204 (12)	196/204 (8)
										196/212 (7)
										212/204/196 (1)
										212/204/188 (1)
Control -16 °C	*Spl-106*	234/270	234/270	260	313	313/243	286/278	270/313 (16)	270/313 (14)	286/260 (16)
								234/313 (14)	234/313 (9)	278/260 (14)
									270/243 (4)	
									234/243 (3)	
	*Spl-163*	188/204	188/204	188/196	188/204	188/204	204/212	188/204 (10)	188/204 (10)	188/204 (8)
								188/188 (9)	188/188 (9)	188/212 (7)
								204/204 (11)	204/204 (11)	196/204 (8)
										196/212 (7)

## Data Availability

The authors declare that the data of this research are not deposited in an official repository and data will be available upon reasonable request.
